# Advantages and Limitations of the Use of Optogenetic Approach in Studying Fast-Scale Spike Encoding

**DOI:** 10.1371/journal.pone.0122286

**Published:** 2015-04-07

**Authors:** Aleksey Malyshev, Roman Goz, Joseph J. LoTurco, Maxim Volgushev

**Affiliations:** 1 Department of Psychology, University of Connecticut, Storrs, Connecticut, United States of America; 2 Institute of Higher Nervous Activity and Neurophysiology, Russian Academy of Sciences, Moscow, Russia; 3 Department of Physiology and Neurobiology, University of Connecticut, Storrs, Connecticut, United States of America; University of Southern California, UNITED STATES

## Abstract

Understanding single-neuron computations and encoding performed by spike-generation mechanisms of cortical neurons is one of the central challenges for cell electrophysiology and computational neuroscience. An established paradigm to study spike encoding in controlled conditions *in vitro* uses intracellular injection of a mixture of signals with fluctuating currents that mimic in vivo-like background activity. However this technique has two serious limitations: it uses current injection, while synaptic activation leads to changes of conductance, and current injection is technically most feasible in the soma, while the vast majority of synaptic inputs are located on the dendrites. Recent progress in optogenetics provides an opportunity to circumvent these limitations. Transgenic expression of light-activated ionic channels, such as Channelrhodopsin2 (ChR2), allows induction of controlled conductance changes even in thin distant dendrites. Here we show that photostimulation provides a useful extension of the tools to study neuronal encoding, but it has its own limitations. Optically induced fluctuating currents have a low cutoff (~70Hz), thus limiting the dynamic range of frequency response of cortical neurons. This leads to severe underestimation of the ability of neurons to phase-lock their firing to high frequency components of the input. This limitation could be worked around by using short (2 ms) light stimuli which produce membrane potential responses resembling EPSPs by their fast onset and prolonged decay kinetics. We show that combining application of short light stimuli to different parts of dendritic tree for mimicking distant EPSCs with somatic injection of fluctuating current that mimics fluctuations of membrane potential in vivo, allowed us to study fast encoding of artificial EPSPs photoinduced at different distances from the soma. We conclude that dendritic photostimulation of ChR2 with short light pulses provides a powerful tool to investigate population encoding of simulated synaptic potentials generated in dendrites at different distances from the soma.

## Introduction

Understanding the principles underlying transformation of synaptic inputs, expressed as membrane potential fluctuations, into the main language used by neuronal ensembles in the brain for communication—sequences of action potentials—is one of the major goals of modern neuroscience. An established paradigm to study this transformation in controlled conditions of in vitro experiments is to record membrane potential and spike responses of neurons to injection of a mixture of signals and fluctuating background currents through the recording electrode. It has been demonstrated using this method that populations of layer 2/3 and layer 5 pyramidal neurons from the neocortex can change their firing rate in response to step or EPSC-like changes of the input very fast, within few milliseconds [1–3], and phase-lock their population firing to signal frequencies up to several hundred Hertz [2,4–7]. Moreover, recent research [7] has confirmed an earlier theoretical prediction that the ability of neurons to detect small abrupt changes in membrane potential crucially depends on the fast dynamics of action potential initiation [8,9]. One drawback of current injection paradigms for studying neuronal encoding is that direct current injection through the recording electrode is technically difficult except at the soma, and the vast majority of synaptic inputs change membrane conductance not in the soma but in the dendrites. To some extent, this drawback could be resolved using dendritic recording, e.g. [10,11], but these methods are typically restricted to large dendrites, and would be difficult to apply to multiple sites in the same neuron. Recent progress in optogenetics provides an opportunity to circumvent this limitation. Boyden et al. [12] demonstrated that photoactivation of cation permeable Channelrhodopsin2 (ChR2) can provide precise, millisecond time-scale control of the membrane potential and spiking in genetically targeted neurons. Thus, transgenic expression of light-activated ChR2 should allow to induce controlled changes of membrane conductance even in thin dendrites, and thus to mimic synaptic inputs to distant dendrites. Here we have experimentally tested this approach and compared it to the current injection through the intracellular recording microelectrode. ChR2 was expressed in pyramidal neurons of layer 2/3 of mouse cortex using in utero electroporation. We show that optogenetics approach provides a useful extension of the tools to study neuronal encoding, but it has its own limitations. Optically induced fluctuating currents have a low cutoff (~70Hz), because ChR2 activation produces only inward currents thus mimicking only excitatory inputs, and ChR2 inactivation has relatively slow kinetics. This limitation could be circumvented by using short (2 ms) light stimuli which produce membrane potential responses resembling EPSPs by their fast onset and a prolonged decay kinetics. We show that combining application of short light stimuli to different parts of dendritic tree for mimicking distant EPSCs with somatic injection of fluctuating current that mimics fluctuations of the membrane potential in vivo, allows to study fast encoding of artificial EPSP photoinduced at different distances from the soma.

## Methods

All experimental procedures used in this study were in accordance with National Institutes of Health regulations. Experimental protocols were approved by the Institutional Animal Care and Use Committee of University of Connecticut.

### In utero electroporation

In utero electroporation was performed as previously described [13, 14]. Briefly, mice were anesthetized for the surgery with a mixture of ketamine/xylazine (100/10 mg/kg i.p.). After the surgery, metacam analgesic was administered for 2 days, at daily dosage of 1 mg/kg s.c. We electroporated plasmids encoding mRFP (pCAG-mRFP,1.5 mg ml–1) to fluorescently label transfected cells along with plasmids expressing ChR2 (1.5 mg/ml). Channelrhodophsin plasmid (pcDNA3.1hChR2-EYFP) was a gift from K Diesseroth, Stanford University, Stanford, CA, and was subcloned into the pCAG plasmid and used before [15]. Electroporation was performed at embryonic day 13–15. During surgery, the uterine horns were exposed and one lateral ventricle of each embryo was pressure injected with 1–2 μl of plasmid DNA. Injections were made through the uterine wall and embryonic membranes by inserting glass microelectrodes (Drummond Scientific) into the lateral ventricle and applying pressure pulses delivered with a Picospritzer II (General Valve). Electroporation was accomplished with a BTX 8300 pulse generator (BTX Harvard Apparatus) and BTX tweezertrodes. A voltage of 40–50 V was used for electroporation.

### Preparation of acute brain slices

Acute brain slices were performed as previously described [16]. Briefly, P28–40 mice were deeply anesthetized with isoflurane. The mouse was decapitated and the brain was rapidly removed and immersed in ice-cold oxygenated dissection buffer containing (in mM): 83 NaCl, 2.5 KCl, 1 NaH_2_PO_4_, 26.2 NaHCO_3_, 22 D-glucose, 72 sucrose, 0.5 CaCl_2_, and 3.3 MgCl_2_. Coronal slices (400 mm) were cut using a vibratome (VT1200S, Leica), incubated in dissection buffer for 30–45 min at 34°C, and then stored at room temperature.

### Electrophysiology

Recordings were made in a submerged-type recording chamber. Recording medium contained (in mM) 125 NaCl, 2.5 KCl, 2 CaCl_2_, 1 MgCl_2_, 1.25 NaH_2_PO_4_, 25 NaHCO_3_, 25 D-glucose and was bubbled with 95% O_2_ and 5% CO_2_. All recordings were made at 28–32°C. The temperature in the recording chamber was monitored with a thermocouple positioned close to the slice, 2–3 mm from the recording site. Whole-cell recordings with patch electrodes were made from RFP-expressing layer 2/3 pyramidal neurons, selected under visual control using RFP-fluorescence, Nomarski optics and infrared videomicroscopy. The patch electrodes were filled with a potassium gluconate-based solution (130 mM potassium gluconate, 20 mM KCl, 4 mM Mg-ATP, 0.3 mM Na_2_-GTP, 10 mM sodium phosphocreatine, 10 mM Hepes) and had a resistance of 4–6 MΩ. Recordings were made with a Dagan BVC-700A (Dagan Corporation, USA) amplifier in the bridge mode. After amplification and low-pass filtering at 10 kHz, data were digitized at 20 kHz and fed into a computer using Digidata 1440A interface and PCLAMP software (Molecular Devices). Fluctuating current for injection into a neuron, ση(t), was synthesized to mimic the effect produced in the soma by numerous balanced excitatory and inhibitory synaptic inputs [17]. η(t) was an Ornstein–Uhlenbeck process with zero mean, unit variance and correlation time τ_I_ = 5 ms, and σ was the standard deviation of the resulting background current noise, scaled to achieve membrane potential fluctuations of ~15–20 mV in amplitude. Membrane potential fluctuations produced by the injected current were similar to those recorded in neocortical neurons in vivo [17–20]. Synaptic transmission was blocked by adding 25 μM 2R-amino-5-phosphonopentanoate (APV), 5 μM 6,7-dinitroquinoxaline-2,3-dione (DNQX) and 80 μM picrotoxin (PTX) to the extracellular solution. Chemicals were obtained from Sigma-Aldrich or Tocris.

### Optical stimulation

A collimated light emitting diode (LED) with output power 650mW and peak emission wavelength of 470 nm (Thorlabs, Newton, New Jersey, USA) was mounted on the epi-illumination port of Olympus BX microscope. Light was passed through 40x Olympus objective. Field diaphragm in the epi-illumination pathway of the microscope was fully closed to achieve locality of stimulation. Analog output of Digidata 1440 ADC (Molecular Devices, USA) was fed either to the modulation input port of LED driver, or to the current input port of Dagan amplifier. Fluctuating voltage, generated by ADC output produced either fluctuation of light intensity, or fluctuation of the current passed through the cell membrane in experiments with intracellular current injection. For optically induced artificial EPSCs 2ms rectangular voltage pulse was applied to the modulation input port of LED driver.

## Results

### Photostimulation of Channelrhodopsin2 expressing neurons

**Fig 1:**
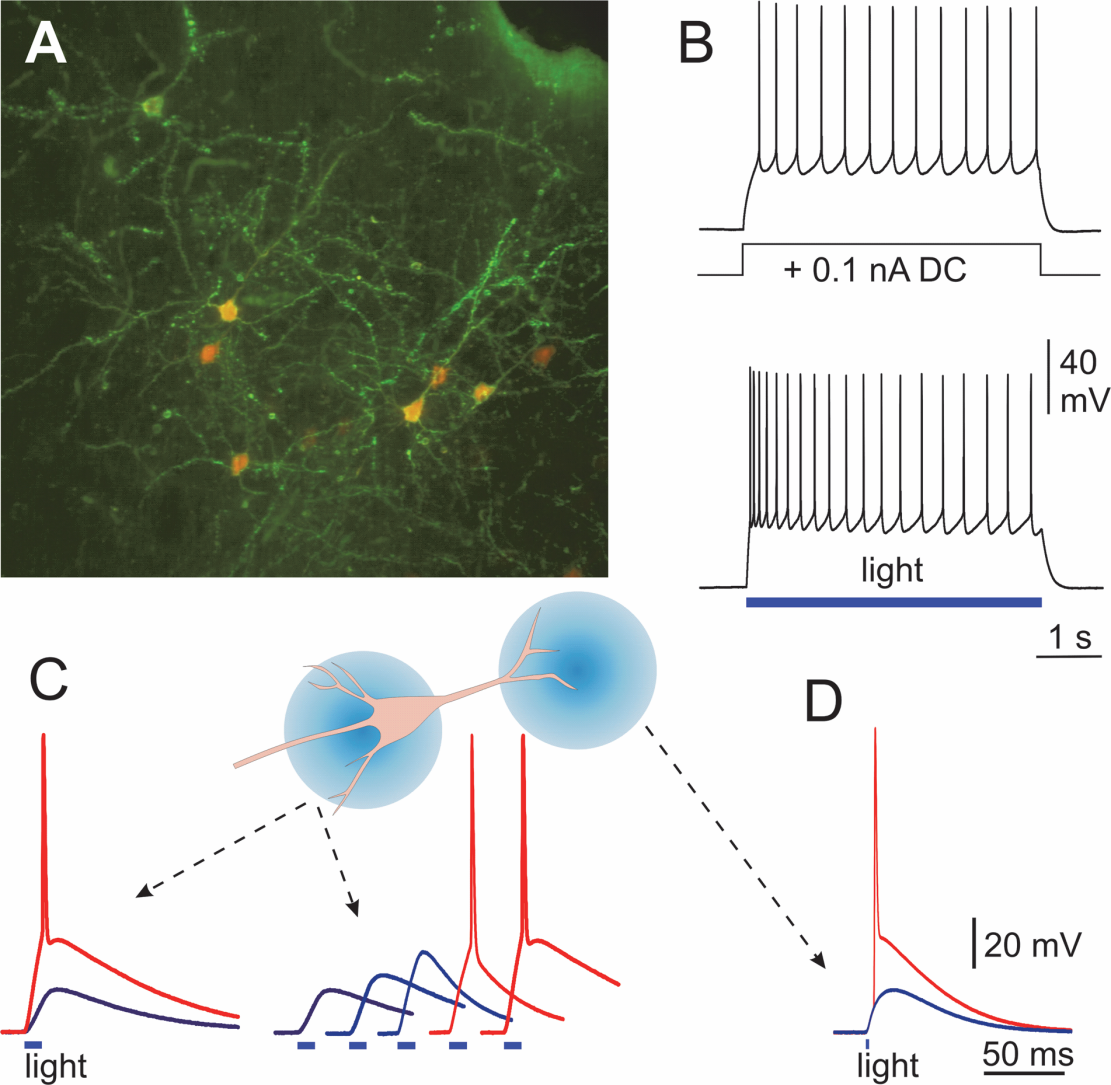
Photostimulation of different parts of a cell expressing channelrhodopsin-2. **A**: Confocal image of mouse cortical neurons expressing ChR2. Mice were transfected *in utero* using intraventricular electroporation with two plasmids, one containing red fluorescent protein that stains cytoplasm and is best visible in cell bodies, and another one containing ChR2 fused with Venus (green/yellow) that stains cell membrane, best visible in dendrites. **B:** Responses of a layer 2/3 pyramidal neuron expressing ChR2 to a step of depolarizing current injected through the recording electrode or induced by light pulse of a 488 nm laser. **C:** Light-induced responses in a ChR2 expressing neuron to 11 ms light pulses of different intensities illuminating soma region. On the left, responses to subthreshold and suprathreshold light stimuli are superimposed. **D:** Light-induced responses in the same cell to short (2 ms) illumination of distal dendrites at subthreshold intensity and at high light intensity that induced dendritic spike.

Mice were transfected *in utero* with the mixture of two plasmids, one containing red fluorescent protein and another one encoding Channelrhodopsin2 (ChR2). This approach produces high level of co-expression of two vectors in pyramidal neurons of layer 2/3 in the cortex of one brain hemisphere [15]. Red fluorescent protein was mostly seen in the soma and proximal dendrites, allowing us to select for recording cells with high level of target genes expression using green light (532 nm) illumination (Fig 1A). With the use of green light we minimized activation of ChR2, and therefore prevented unnecessary excitation of neurons during cell selection. After establishing whole cell configuration blue-light illumination with 470 nm LED, mounted on the epifluorescent port of Olympus BX microscope, was used for ChR2 activation. Illumination with a long pulse of light produces a prolonged depolarization and repetitive firing, which were similar to those produced by direct injection of depolarization current through the intracellular recording electrode (Fig 1B). The photoinduced responses had, however, slightly different temporal dynamics: membrane depolarization did not remain at the same level throughout the illumination, but showed a slight decay, and frequency of action potentials showed a clear decrease from the beginning toward the end of response. The decrease of the firing rate during photoinduced response was clearly more pronounced than spike adaptation during response to a depolarizing current injection in this neuron. This difference of the dynamics of membrane potential response was also seen with subthreshold responses (data not shown), indicating that it might reflect the kinetics of ChR2 activation [12].

For local photostimulation we used fully closed diaphragm in the epi-fluorescence unit of the microscope. We estimated that with fully closed field diaphragm illumination through a 40x objective produced a spot of light of an approximate diameter of 80 μm. By centering this spot over the soma or over the distal portion of the apical dendrite we were able to evoke in the neuron clearly different responses. Illumination of the somatic region with 11-ms light pulse from a 470 nm LED produced short-lasting depolarization response (Fig 1C). Increasing the intensity of light led to a proportional increase of the amplitude of depolarization, eventually reaching the spike threshold. Supra-threshold stimulation reliably induced action potentials (Fig 1C). Brief light stimuli (2 ms) applied to distal part of the apical dendrite also produced depolarization response in recorded cell. When light intensity exceeded the threshold level, distally applied stimuli could induce dendritic action potentials, confirming the locality of stimulation (Fig 1D). Depolarization responses produced by very short light pulses had the time-course very similar to that of synaptically induced EPSPs. These were purely light-induced responses, because excitatory and inhibitory synaptic transmission was blocked with DNQX, APV and PTX in these experiments, excluding the possibility of contamination of the light induced responses by synaptic inputs even at highest light intensities used. Thus, short-pulse photoactivation of ChR2 can be used to produce EPSP-like depolarization at distal parts of the dendritic tree, and to study how conductance changes that occur at distal dendrites are encoded in spike responses of cortical neurons.

### Membrane potential responses to photoinduced fluctuating currents

**Fig 2:**
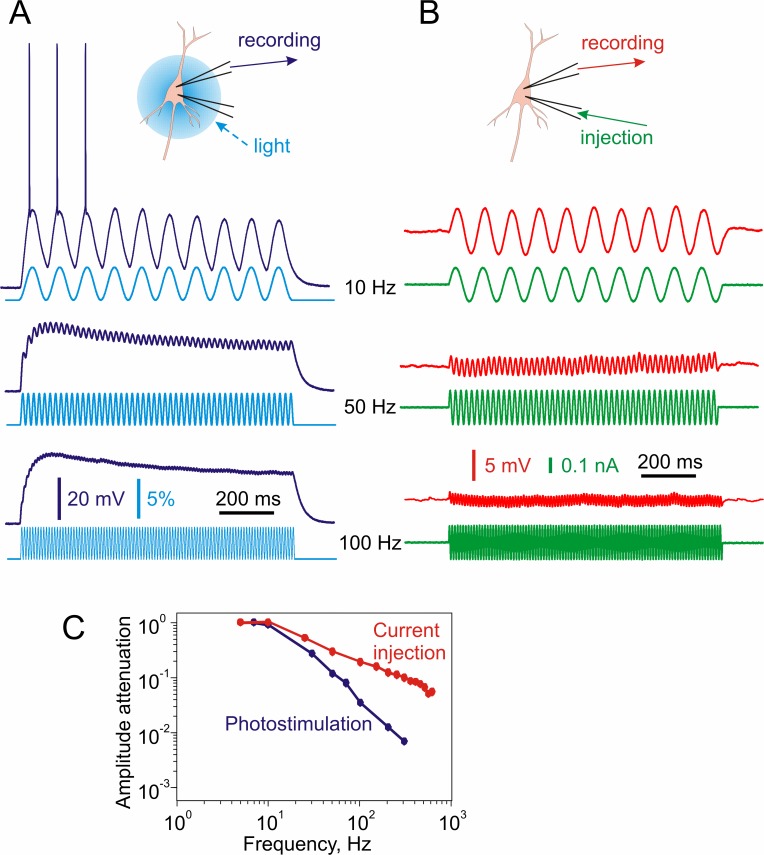
Membrane potential responses to sine-wave modulation of light intensity express stronger attenuation of high frequencies than responses to intracellular current injection. **A:** Membrane potential responses (blue traces) of a layer 2/3 pyramidal neuron expressing ChR2 to a 488 nm diode light modulated by sine-wave signals at 10, 50 and 100 Hz (light blue traces). The amplitude of the sine-wave modulation of light intensity was kept constant. **B:** Membrane potential responses (red traces) of a neuron to injection of sine-wave current at 10, 50 and 100 Hz (green traces). Two-electrode experiment. One electrode was used for current injection, and another electrode for recording of membrane potential responses. The amplitude of the sine-wave current was kept constant. **C:** Attenuation of the amplitude of membrane potential modulation in responses to sine-wave signals of different frequencies injected through the intracellular electrode or induced by photostimulation.

We asked whether photostimulation of ChR2-expressing neurons can be used to mimic the effect of synaptic bombardment produced by activity at numerous inputs to a neuron in vivo [17], and to study how changes in membrane conductance in different areas of dendritic tree are transferred into changes of neuronal firing rate. So far, this transformation has been studied using injection of fluctuating currents in the soma [2,4–7], which does not take into account possible dendritic filtering of input signals. To determine temporal precision of control over the membrane potential fluctuations by photostimulation, we first used the most direct and clear-cut approach: recording of membrane potential response to photostimulation of ChR2-expressing neuron with light, modulated by sine wave of different frequencies. As a reference we used double-electrode patch experiments in which sine wave current was injected through one electrode and subthreshold membrane potential response was recorded via the second intracellular electrode. Double-electrode recording scheme was chosen in order to diminish possible artefacts associated with current injection and recording through the same electrode in single electrode current clamp mode, such as possible distortions introduced by non-ideal bridge compensation. Low frequency oscillations (below 10 Hz) induced by both photostimulation and direct current injection where transferred into membrane potential oscillation equally well (Fig 2A and 2B). However, starting from ~ 50 Hz and at higher frequencies attenuation of light-induced sine-wave signals became significantly stronger than of those induced by intracellular current injection. In Fig 2 example, membrane potential responses to photostimulation were attenuated 5.4 times stronger than current injection-induced at 100Hz, 9.7 times stronger at 200Hz, and 14.1 times stronger at 300Hz (Fig 2C). High frequency photostimulation also induced a pronounced depolarization shift of the membrane potential because ChR2 activation produced net inward current which mimicked only excitatory but not inhibitory input. This fact, together with relatively slow kinetics of ChR2 inactivation, might be the main reasons for the strong attenuation of high frequency sine wave signals.

**Fig 3:**
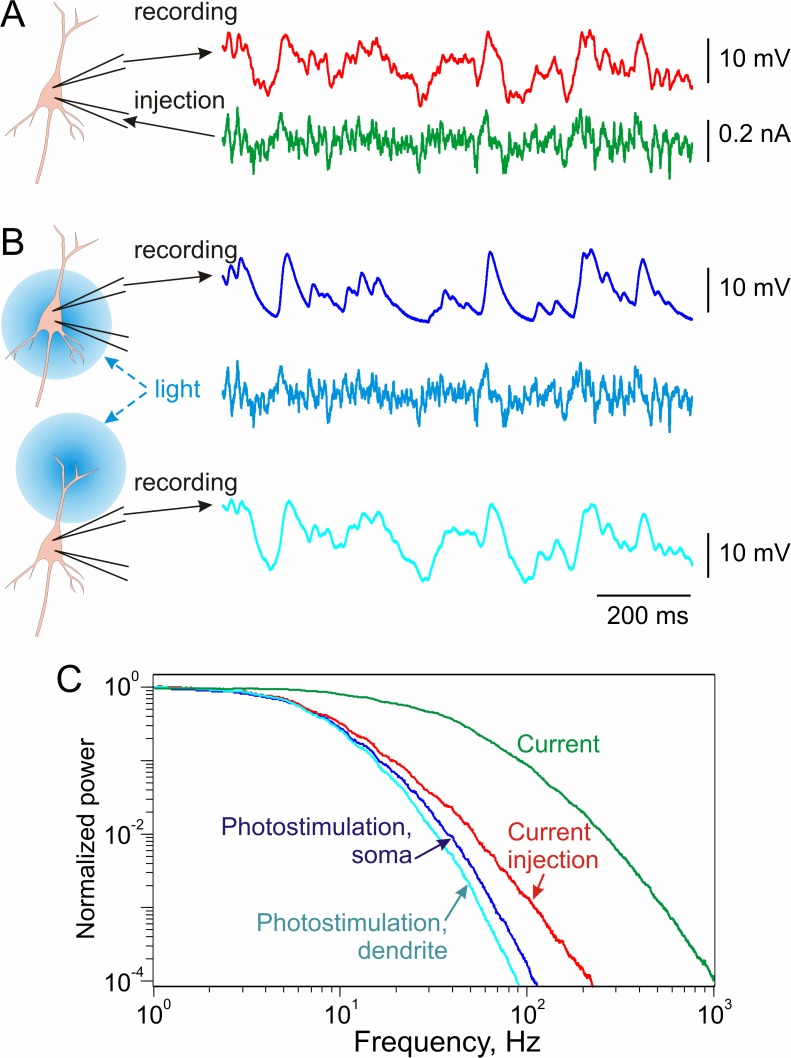
Frequency response of the membrane potential to fluctuating current injected through intracellular electrode or photoinduced in somatic region and in distal dendrites. **A:** Membrane potential responses of layer 2/3 pyramidal neuron to injection of subthreshold fluctuating current through intracellular electrode. Two-electrode experiment. **B:** Membrane potential responses of the same neuron to illumination of somatic region or distal dendrites by the light from 488 nm diode with intensity modulated by the same fluctuating current as used for intracellular injection. The illumination field for dendritic stimulation was shifted ~150 μm from the soma. For dendritic stimulation light intensity was increased 2.5 times in order to induce membrane potential fluctuations of the the same amplitude range. **C:** Normalized power spectra of the current used for injection or modulation of light intensity, and of membrane potential responses to intracellular current injection and to photostimulation of the somatic region and of distal dendrites.

Experiments with sine-wave modulation clearly demonstrated significantly stronger attenuation of high frequencies during ChR2 photostimulation as compared to current injection through the intracellular electrode. Sine wave method, however, produces only point-wise characterization of attenuation at different frequencies since the number of frequencies which could be studied in one experiment is limited. To systematically characterize attenuation of currents with realistic frequency composition, we modulated the intensity of the blue light used to activate ChR2 with fluctuating noise current (correlation time constant τ_I_ = 5 ms) and compared the power spectra of the injected noise and the induced membrane potential fluctuations. In this series of experiments we applied photostimulation not only to the somatic region of ChR2-expressing neurons, but also to distal parts of their dendritic arbor (Fig 3A and 3B). Power spectra of membrane potential responses to the fluctuating current-modulated photostimulation were compared to the power spectrum of membrane potential responses to injection of the same fluctuating current through the intracellular electrode, and to the power spectrum of the current used for injection or modulation (Fig 3C). This approach revealed several important features of the signal filtering. First, even current directly injected into the cell through intracellular electrode is subject to strong, frequency-dependent low-pass filtering by neuronal membrane (Fig 3C, green and red curves). Second, photostimulation of the somatic area of the same neuron with the light modulated by the same fluctuating current revealed additional filtering (Fig 3C, blue curve) that might be introduced by the activation and inactivation kinetics of ChR2. Third, photostimulation of the distal portion of the apical dendrite 150 mm away from the soma (in layer 1 area) with the same light pattern produced depolarization of a smaller amplitude than somatic photostimulation. To facilitate comparison of the responses to the somatic and distal photostimulation, we have increased the light intensity for stimulation of distal dendrites to achieve the same magnitude of membrane potential fluctuations as those produced by photostimulation of the somatic region. Despite the prominent high frequency filtering introduced by the ChR2 kinetics, these experiments revealed that signals arriving at the distal dendrites are subject to additional filtering imposed by dendritic membrane. Indeed, power spectrum of membrane potential fluctuations induced by distal photostimulation contained less high frequency components than power spectrum of membrane potential response to somatic photostimulation (Fig 3C, blue and cyan curves).

### Phase-locking of spiking to photoinduced fluctuating currents

**Fig 4:**
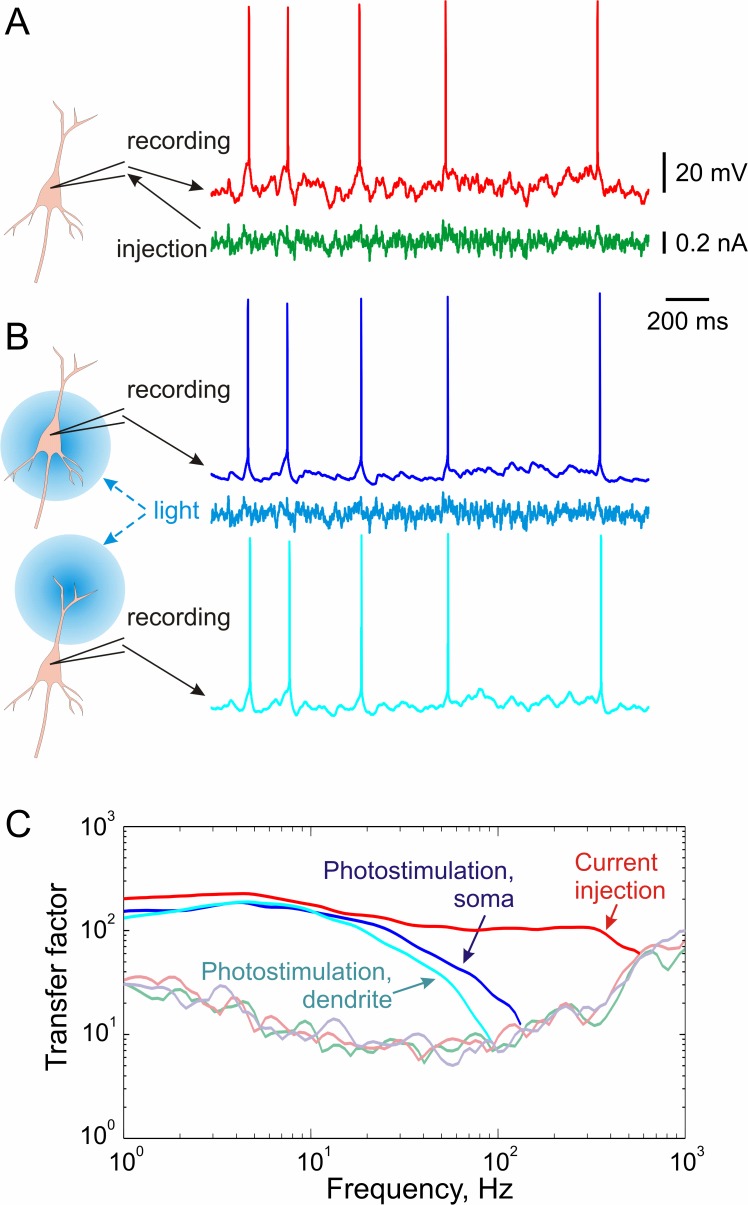
Frequency transfer functions calculated from responses of a neuron to fluctuating current, either injected through intracellular electrode or photoinduced in somatic region and in distal dendrites. **A:** Membrane potential and spike responses to intracellular injection of fluctuating current. DC current was added to maintain target firing rate of ~5 Hz. **B:** Membrane potential responses to illumination of the somatic region or distal dendrites by light modulated by the same fluctuating current as used for intracellular injection. DC current was injected through the somatic electrode to maintain target firing rate. **C:** Frequency transfer functions calculated from responses to fluctuating current, injected intracellularly or photoinduced in the soma or in distal dendrites. Transfer functions were calculated using modified Higgs & Spain [6] method. Faint lines show 95^th^ percentile of N = 500 transfer functions calculated using shuffled spike timings. Transfer factors are considered significant when above the 95^th^ percentile of shuffled-spike values; transfer function is cut at the intersection with the 95^th^ percentile curve [7].

**Fig 5:**
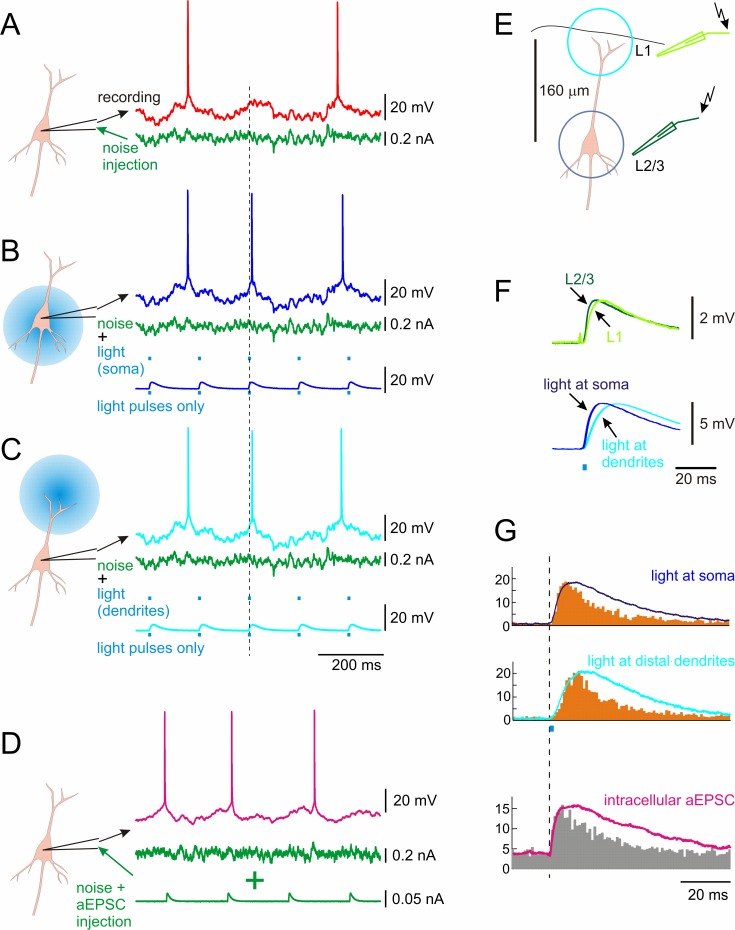
Responses of a layer 2/3 pyramidal neuron to proximal and distal photostimulation, and electrical stimulation of proximal or distal synaptic inputs. **A:** Membrane potential response to fluctuating current injected through the intracellular recording electrode. **B, C:** Membrane potential response to a mixture of intracellular injection of the same fluctuating current and photoinduced optical EPSCs (oEPSCs) produced by 2 ms pulses of 488 nm light illuminating somatic region (B) or distal dendrites (C). Dashed vertical line shows the timing of an oEPSC which led to generation of an additional action potential. Note that other oEPSCs did not lead to additional spikes. **D:** Response of pyramidal neuron to intracellular injection of fluctuating noise current with immersed small artificial aEPSCs. **E:** A scheme of optical and electrical stimulation of a layer 2/3 pyramidal neuron. Black line on top shows cortical surface. Electrical stimuli were applied through patch stimulation electrodes located in layer 1 or in layer 2/3. For optical stimulation, either somatic region or distal part of apical dendrite was illuminated, as indicated by the circles. **F:** EPSCs evoked by electrical stimulation and photoinduced oEPSCs. All responses were recorded by intracellular pipette in the soma. Note that EPSCs induced by electrical or optical stimulation in layer 1 have slower onsets, indicative of their more distal origin, than EPSCs induced by electrical or optical stimulation in layer 2/3. **G:** PSTHs of spike responses to EPSCs immersed in fluctuating current. EPSCs were photoinduced in the somatic region or in distal dendrites, or produced by intracellular current injection. Note that in all three cases, the increase of the firing rate follows the dynamics of EPSC onset. Abscissa is population firing rate in Hz.

Next, we asked how membrane potential fluctuations induced by photostimulation or by intracellular current injection are transformed into changes of neuronal firing. We measured frequency response function of neurons using spike responses to fluctuating current, injected through the microelectrode or induced by photostimulation of the different parts of neuron. In these experiments, the current intensity was adjusted to produce membrane potential fluctuations of ~15–20 mV, which is typical range observed in vivo [17–20]. Additional DC current was used to achieve target firing rate of 3–6 Hz. In these experiments the quantitatively measured parameter of neuronal response was the timing of action potentials. We did not quantify membrane potential fluctuations, which were contaminated by action potentials anyhow. Therefore single-electrode recordings were used in experiments presented in Figs 4 and 5. Fig 4A and 4B shows examples of cell responses to injection of fluctuating currents and to photostimulation with light intensity modulated with the same current. Frequency response function was calculated using modified method of Higgs and Spain [6], as the ratio of Fourier transforms of spike-triggered average and autocorrelation of the injected current, with frequency-dependent windowing used to improve the signal-to-noise ratio (for details see [7]). Frequency response function measured with somatic current injection through the microelectrode exhibits high cut-off frequency around 300–400 Hz (Fig 4C, red curve), consistent with previous reports for pyramidal neurons from layer 2/3 [2,6,7] and layer 5 of the neocortex [4,5]. However the cutoff of the frequency response function measured using photostimulation of the somatic region was significantly lower, at frequencies around 50 Hz (Fig 4C, blue curve). Spike responses to photocurrents induced at distal dendrites showed an even lower cut-off frequency than responses to somatically induced photocurrents (Fig 4C, cyan curve). The low cutoff frequency of responses to photostimulation-induced fluctuations might be due to a strong attenuation of high frequency components in light-induced fluctuations due to a slow inactivation kinetics of ChR2 (Fig 3). Thus, our results demonstrate that the use of amplitude-modulated ChR2 photostimulation leads to a dramatic underestimation of the cut-off frequency of spike responses. Because of the strong attenuation of high frequency components due to a slow inactivation kinetics of ChR2 (Figs 2 and 3), light-induced fluctuations of the membrane potential do not reproduce the whole frequency range of fluctuations observed in vivo. This imposes severe limitations on the use of ChR2-photostimulation for studying frequency response function of cortical neurons using photoinduced fluctuating currents.

### Fast spike responses to artificial optically induced EPSCs

How to circumvent this limitation? As we have noted above, very short (2 ms) light stimuli, as typically used for inducing single spikes in optogenetic experiments, produced membrane potential responses with kinetics closely resembling EPSPs, when light intensity remained subthreshold (Fig 1D). The ascending phase of these optically induced EPSP (oEPSP) is determined by the fast activation of ChR2 (time constant <2 ms, [21]), while a slower decay reflects slow inactivation of ChR2 (time constant 9.2 ms, [21]). Indeed, the ascending phase of oEPSC induced by illumination of somatic region had time constant of 1.4 ms, producing an oEPSP with time constant of the rising phase of 3.7 ms (Fig 5F). Note that because oEPSPs have a slow decay, which is determined by slow time course of channel inactivation and time constant of the cell membrane, power spectrum of oEPSPs (not shown), while having more high frequencies, is still dominated by low frequency components and the cut-off frequency of membrane potential response to short pulses remains similar to that of the response to noise-modulated photostimulation. However the fast onset of the oEPSPs opens an opportunity to study fast components of neuronal responses photoinduced at distant dendritic locations. To explore this possibility we applied photostimulation to somatic region and to the distal portion of the apical dendrite, and compared the induced oEPSPs to EPSPs evoked by electric stimulation applied near the soma (in layer 2/3) and in layer 1, lateral to the region of apical dendrite arborization. The ascending phase of responses photoinduced in somatic region was steeper compared to oEPSPs induced by photostimulation of distal dendrites (Fig 5E). The 10–90 rise time (the time required for the response to rise from 10% to 90% of its amplitude) for somatically induced oEPSPs was 4 ms vs. 8 ms for oEPSPs induced by stimulation of apical dendrites. In these experiments, the intensity of distal photostimulation was increased to achieve the same oEPSP amplitude as with somatic stimulation. It is known that in layer 5 pyramidal neurons EPSP rise time is longer for synaptic inputs located at more distant dendrites [22,23]. To reproduce this situation for layer 2/3 pyramidal neurons we used local extracellular stimulation via low-resistance patch pipettes, one positioned within ~50 μm from the cell soma and the other one in layer I approximately 200 μm laterally from the apical dendrite axis (Fig 5D). Amplitude and duration (range 50–200 μs) of electrical stimuli at both stimulation sites were adjusted to evoke EPSPs of ~2 mV amplitude, constant and stable latency and no visible polysynaptic components. EPSPs evoked by near-somatic and distant stimulations had clearly different rising phase kinetics (Fig 5E). The electrode located close to the soma evoked EPSP with average 10–90 rise time of 2.6 ms, while layer 1 stimulation electrode elicited EPSP with rise time of 5 ms. Thus, for both the light-induced oEPSPs and for the electrically evoked EPSPs, responses from distant dendrites had an approximately twice longer rise time than responses from perisomatic region. This similarity of the rise time increase indicates that distal responses, induced optically or electrically, were subject to similar degree of filtering during their propagation to the soma, and thus originated at similar distances from the soma.

Now we can use photostimulation to address the question how proximal and distal oEPSPs are encoded in firing rate responses of neuronal populations. We used a modification of established paradigm to study encoding, in which artificial EPSCs are immersed in fluctuating noise [1–3]. This approach is based on the assumption that N presentations of the same test signal (here oEPSP) immersed in different realizations of fluctuating noise to one cell provides an estimate of the response of a population of N neurons, each receiving the test oEPSP on the background of individual pattern of activity at its other inputs. Fluctuating current producing *in vivo*-like membrane potential fluctuations was injected intracellularly through the recording electrode. DC current was added to achieve target firing rate of 3–5 Hz. Short light stimuli inducing oEPSPs were applied every 150 ms to the soma region or to the region of branching of the apical dendrite in layer 1. Fig 5A–5C shows an example of such experiment. Addition of oEPSPs to the fluctuating noise could induce additional action potential (vertical dashed line) that was not present in responses to noise only injection. In the Fig 5B and 5C example, additional spike is evoked by oEPSPs induced either in the somatic region or at distal portion of the apical dendrite. In most cases, however, oEPSPs did not induce any visible changes in on-going spiking activity. The peristimulus time histograms constructed using spike responses to 3000 presentations of oEPSP shows clear oEPSC-produced peaks. Notably, the shape of the rising front of population response corresponds to the rising phase of the oEPSP. This holds both for responses to oEPSPs induced by somatic stimulation with a faster onset dynamics, as well as for the responses to distally induced oEPSPs, which had a slower onset.

Data from these experiments with photoinduced oEPSCs presented on the background of fluctuating current do not allow for calculation of the transfer function, because, first, the membrane potential fluctuations are dominated by the fluctuating current injected via the intracellular electrode, and second, the waveforms of the oEPSCs were not contained in the input signal. Therefore to compare the dynamics of population responses to oEPSCs with the dynamics of spike responses to intracellularly injected aEPSCs we performed experiments similar to those described in Fig 5D and 5C, but with electrical aEPSCs injected intracellularly instead of optically induced oEPSCs. Fig 5G illustrates high degree of similarity between spike responses to optically induced oEPSPs and to intracellularly injected aEPSCs: PSTHs of both responses are fast, with the onset dynamics that closely follows the rising phase of the membrane potential response. Thus, the use of photoinduced oEPSPs combined with intracellular injection of fluctuating current mimicking background synaptic activity, provides useful tool to study how EPSP-like conductance changes at different potions of dendritic tree are encoded into changes of neuronal firing rates.

## Discussion

Here we show that photostimulation of neurons expressing ChR2 can be used to evoke conductance changes localized to different parts of the dendritic tree. This makes optogenetic approach a useful tool to study how changes of conductance, mimicking synaptic inputs at different parts of dendritic tree, are encoded in spike responses. However, it was not possible to reproduce rapid, in vivo like fluctuations of the membrane potential using ChR2 photostimulation, because frequencies above ~70Hz were severely attenuated because activation of ChR2 could mimic only excitatory inputs, and their inactivation has relatively slow kinetics. This led to a dramatic underestimation of the ability of neurons to phase lock their firing to high frequency components of input signals. We further show that this limitation can be circumvent by combining current injection via the recording electrode for mimicking in vivo-like membrane potential fluctuations with photoinduction of optical EPSPs at different parts of dendritic tree.

### Temporal resolution of photoinduced responses

Light activated cation channel ChR2 has been used in a variety of experimental paradigms for activating single neurons and neuronal populations to study their role in operation of neural networks. Fast activation dynamics of ChR2 allows induction of action potentials in genetically targeted neurons with millisecond precision using short (few ms) light stimuli [12]. However, during repeated stimulation with a series of short pulses of light action potentials could be induced reliably only up to 10–20 Hz [12,21,24] or 40–50 Hz [25].

The limiting factor for reliable induction of spikes at higher frequencies is relatively slow inactivation of ChR2, which has off-kinetics time constant of 9.2 ms [21]. The low-pass filtering effect of slow ChR2 inactivation stands out clearly in membrane potential responses to photostimulation with amplitude-modulated light. Our experiments with sine-wave and fluctuating current modulated photostimulation show severe attenuation of frequencies above ~50–60 Hz in photoinduced responses. These results are in agreement with prior reports on frequency limits of ChR2 photoactivation [12,21,24,25], and with results of a recent study that combined computational analysis of ChR2 kinetics using Markov model with electrophysiological analysis of photoinduced currents in dissociated cultures of cortical neurons [26]. Both modelling and electrophysiological results of Tchumatchenko et al [26] show that photostimulation of ChR2 or a faster E123A (ChETA) mutant [21] allows membrane potential modulation at frequencies up to ~70–100 Hz. This range corresponds to our results from ChR2-expressing pyramidal neurons from layer 2/3 in slices from mouse neocortex.

To what extent can photostimulation of ChR2 expressing neurons reproduce “high-conductance” state of neurons in vivo, which is produced by on-going synaptic bombardment, and characterized by irregular firing and low input resistance due to massive activation of synaptic conductance [17]? Clear advantage of the photostimulation over current injection through somatic intracellular electrode is that opening of ChR2 can increase peripheral conductance, much like distally located excitatory synapses would do. The resulting profile of photoinduced currents over the dendritic tree might correspond more closely to the profile of synaptically induced excitation, with stronger conductances and currents in the dendrites than in the soma. In contrast, currents injected through somatic electrode produce transmembrane currents that are strongest at the soma, but decay toward dendrites. However, optogenetic approach has two serious limitations. The first is that ChR2 activation can reproduce only excitatory conductance, while in high conductance state in vivo excitation and inhibition are dynamically balanced [27–30]. Second limitation is that photoinduced currents reproduce only low-frequency portion of the dynamic range of membrane potential fluctuations of real neurons. Low-pass filtering of photoinduced membrane potential fluctuations leads to reduced ability of neurons to phase lock their firing to high frequency components of the input, and thus to encode rapidly changing input signals. Indeed, our results show that while in response to photoinduced currents neurons could not phase-lock their firing to frequencies above ~100 Hz, in response to current injection through intracellular electrode these same neurons could phase-lock their firing to several-fold higher frequencies, up to ~400–500 Hz. These results for layer 2/3 pyramidal neurons from mouse neocortex are consistent with prior studies showing that layer 2/3 and layer 5 pyramids from rat cortex in vitro and cat visual cortex neurons in vivo can reliably phase-lock their firing to signal frequencies up to 300–600 Hz [2,4–7]. Therefore we conclude that optical stimulation of ChR2 expressing neurons with modulated light cannot reproduce the whole dynamic range of frequency response of cortical neurons, and thus cannot be used as substitute of injection of fast fluctuating current through recording electrode in studies of neuronal coding of fast signals.

If slow inactivation kinetics of ChR2 is the main factor limiting frequency response of neurons to noise-modulated photoinduced currents, a logical way around this limitation would be to use faster channels, such as E123A (ChETA) mutant [21], which has activation and inactivation kinetics about 2 times faster than wild type ChR2 ([21]: flash to peak latency 0.8 ms vs. 2.4 ms, and off kinetics time constant 4.0 ms vs 9.2 ms). Interestingly, however, recent study [26] reports that the use of fast E123A mutant brought about only minor improvement of the dynamic range. The range of light modulation frequencies that could support significant photocurrents increased by only few Hz, from 69 Hz in neurons expressing wild-type ChR2 to 73 Hz in neurons expressing E123A mutant channel [26]. We interpret these results as indicative of the crucial role for excitatory-inhibitory balance and coordinated activity of excitatory and inhibitory inputs in generation of high frequency fluctuations in cortical neurons. Thus for now, somatic injection of fluctuating current or dynamic clamp reproducing fluctuating conductance in the soma remain the only technically feasible ways for reproducing controlled high frequency membrane potential fluctuations in vitro. Complete replication of in-vivo like high frequency fluctuations of the membrane potential using photoactivation in vitro would require expression of two channels activated by different wavelength and supporting excitatory and inhibitory conductance. Recently, technical tools necessary for such experimental setting are starting to emerge [31] making such experiments potentially possible in the future.

### Local photostimulation at distant dendritic sites

Photostimulation of ChR2 expressing neurons makes possible to induce conductance changes localized to different parts of the dendritic tree, and thus to study how distant inputs are encoded in spike responses. For this purpose, spatial resolution of ChR2 photoactivation and possibility for separate activation of different regions of the neuron is important. Prior research has shown that in cultured cortical neurons high spatial resolution (~30–60 μm) could be achieved when low intensity of light was used for stimulation of neurons with high level of ChR2 expression [32]. Our results show that photostimulation can be used to induce local changes of conductance also in layer 2/3 pyramidal neurons in slices. Locality of photostimulation in our experiments is supported by three lines of evidence. First, rise time of oEPSPs induced by short light pulses applied to distant parts of apical dendrite arbor was longer than rise time of oEPSPs induced by the same light pulse, applied to the soma. This difference corresponds well to the difference of rise times of synaptic responses evoked by electrical pulses applied through stimulation electrodes located close to the soma or around apical dendrite in layer 1, as well to prior results from layer 5 pyramids [22,23]. Second, strong photostimulation of distal dendrites produced clearly distinguishable dendritic action potential indicating that at least main activation of ChR2 channels occurred in periphery. Third, membrane potential fluctuations produced by noise-modulated photostimulation exhibited stronger low-pass filtering when applied to distal dendrites as compared to somatic photostimulation. Therefore paradigm of stimulation which we used in our experiments—LED source mounted to epifluorescent port of the microscope with field diaphragm fully closed—produced local photostimulation. The spatial resolution might be improved by using a 60x objective instead of 40x to illuminate a smaller region, and/or by using focused laser beam for photostimulation.

### Outlook: Advantages and limitations of photostimulation for studying encoding

The use of optically induced responses has several advantages over the use of electrically evoked EPSPs for studying how inputs to proximal and distal dendrites are encoded in spike responses. First, photostimulation allows better localization of induced conductance changes and systematic quantitative variation of the locus of conductance change over the dendritic tree. Second, unlike probabilistic synaptic responses, photostimulation induced highly reproducible and stable responses, which is a very important feature when acquisition of thousands of responses is necessary. Third, photostimulation allows precise control over the amplitude and time course of artificial oEPSPs (within the limits imposed by ChR2 kinetics). Finally, responses to photostimulation can be studied with synaptic transmission blocked, which decreases background conductance, and allows more complete control over membrane potential of the cell.

However, as discussed above, photostimulation of ChR2 expressing neurons can reproduce membrane potential fluctuations only up to ~100 Hz, while higher frequency components of the input are severely attenuated. Therefore for studying fast encoding we suggest to combine photoinduced oEPSPs that can mimic postsynaptic responses evoked at different portions of dendritic tree, with intracellular injection of fluctuating current that mimics membrane potential fluctuations in vivo.

This experimental setting can be considered as a model of a pair of monosynaptically connected neurons, with precisely defined amplitude, time course and location of the input signal, and controlled parameters of background activity. This experimental setting allows us to study how input stimuli are encoded in changes of neuronal firing, and how spike encoding depends on parameters of the input signals and patterns of the background activity. Presentation of these stimuli on the background of different realizations of fluctuating noise allows to study population encoding [1–3], and dependence of signal detection on time and population size. We conclude that photostimulation of ChR2 expressing neurons with short light pulses provides a powerful tool in investigation of population encoding of synaptic inputs with strong emphasis on location dependence of encoding.
